# Supplementary material for: Robustness analysis in an inter-cities mobility network: modeling municipal, state and federal initiatives as failures and attacks toward SARS-CoV-2 containment
Source: PeerJ. 2020 Nov 5;8:e10287. doi: 10.7717/peerj.10287 (PMC7649015; doi:10.7717/peerj.10287)
Supplement: Supplemental Information 1 [file peerj-08-10287-s001.pdf]

Supplemental Material

Tables S1, S2, and S3 refer to the list of the 27 Brazilian States (BS) sorted according to their associated network metric under η_0, η_1 and η_2, respectively. In the second column of each Table, the states are ordered according to the date they reported the first COVID-19 patient.

Tables S4, S5, and S6, refer to the list of the first 30 cities of Sao Paulo State (SP) sorted according to their associated metric under η_0, η_1 and η_2, respectively. In the second column, the cities are ordered according to the date they reported the first COVID-19 patient.

Tables S7, S8, and S9, refer to the list of the first 30 Brazilian cities (BR) sorted according to their associated metric under η_0, η_1 and η_2, respectively. In the second column, the cities are ordered according to the date they reported the first COVID-19 patient. The states' acronyms mean the following: Acre (AC), Alagoas (AL), Amapá (AP), Amazonas (AM), Bahia (BA), Ceará (CE), Distrito Federal (DF), Espírito Santo (ES), Goiás (GO), Maranhão (MA), Mato Grosso (MT), Mato Grosso do Sul (MS), Minas Gerais (MG), Pará (PA), Paraíba (PB), Paraná (PR), Pernambuco (PE), Piauí (PI), Rio Grande do Norte (RN), Rio Grande so Sul (RS), Rio de Janeiro (RJ), Rondônia (RO), Roraima (RR), Santa Catarina (SC), São Paulo (SP), Sergipe (SE), Tocantins (TO).

Table S1: List of the 27 Brazilian States (BS) sorted according to their associated metric under η_0. In the second column, the states are ordered according to the date of their first confirmed case.

	COVID-19	s	k	$\mathcal{V}$	b
1	São Paulo	São Paulo	Distrito Federal	Pará	Pará
2	Rio de Janeiro	Paraná	São Paulo	Distrito Federal	Rondônia
3	Bahia	Minas Gerais	Minas Gerais	São Paulo	Acre
4	Distrito Federal	Goiás	Goiás	Minas Gerais	Alagoas
5	Minas Gerais	Bahia	Bahia	Goiás	Distrito Federal
6	Alagoas	Santa Catarina	Pará	Bahia	São Paulo
7	Rio Grande so Sul	Mato Grosso	Mato Grosso	Mato Grosso	Minas Gerais
8	Paraná	Rio Grande so Sul	Rio de Janeiro	Alagoas	Goiás
9	Santa Catarina	Mato Grosso do Sul	Alagoas	Pernambuco	Amazonas
10	Goiás	Maranhão	Pernambuco	Maranhão	Bahia
11	Rio Grande do Norte	Pará	Maranhão	Rio de Janeiro	Mato Grosso
12	Pernambuco	Pernambuco	Paraná	Rondônia	Maranhão
13	Amazonas	Tocantins	Sergipe	Paraná	Pernambuco
14	Sergipe	Rio de Janeiro	Ceará	Sergipe	Paraná
15	Mato Grosso do Sul	Ceará	Tocantins	Tocantins	Tocantins
16	Ceará	Piauí	Rondônia	Ceará	Rio de Janeiro
17	Acre	Rondônia	Mato Grosso do Sul	Mato Grosso do Sul	Sergipe
18	Pará	Paraíba	Rio Grande so Sul	Rio Grande so Sul	Ceará
19	Paraíba	Distrito Federal	Piauí	Piauí	Mato Grosso do Sul
20	Tocantins	Sergipe	Santa Catarina	Santa Catarina	Rio Grande so Sul
21	Espírito Santo	Espírito Santo	Espírito Santo	Espírito Santo	Santa Catarina
22	Piauí	Alagoas	Paraíba	Paraíba	Espírito Santo
23	Mato Grosso	Rio Grande do Norte	Rio Grande do Norte	Rio Grande do Norte	Piauí
24	Rondônia	Acre	Acre	Acre	Paraíba
25	Amapá	Amazonas	Amazonas	Amazonas	Rio Grande do Norte
26	Maranhão	Roraima	Roraima	Roraima	Amapá
27	Roraima	Amapá	Amapá	Amapá	Roraima

Table S2: List of the 27 Brazilian States (BS) sorted according to their associated metric under η_1. In the second column, the states are ordered according to the date of their first confirmed case.

	COVID-19	s	k	$\mathcal{V}$	b
1	São Paulo	São Paulo	Goiás	Bahia	Bahia
2	Rio de Janeiro	Paraná	Bahia	Goiás	Goiás
3	Bahia	Minas Gerais	Mato Grosso	São Paulo	São Paulo
4	Distrito Federal	Santa Catarina	São Paulo	Paraíba	Pernambuco
5	Minas Gerais	Goiás	Mato Grosso do Sul	Mato Grosso	Ceará
6	Alagoas	Bahia	Paraná	Minas Gerais	Paraíba
7	Rio Grande so Sul	Mato Grosso	Minas Gerais	Maranhão	Maranhão
8	Paraná	Mato Grosso do Sul	Santa Catarina	Ceará	Mato Grosso
9	Santa Catarina	Rio Grande so Sul	Pernambuco	Pernambuco	Minas Gerais
10	Goiás	Maranhão	Ceará	Paraná	Paraná
11	Rio Grande do Norte	Pará	Maranhão	Mato Grosso do Sul	Sergipe
12	Pernambuco	Pernambuco	Rio Grande so Sul	Pará	Mato Grosso do Sul
13	Amazonas	Tocantins	Piauí	Piauí	Pará
14	Sergipe	Rio de Janeiro	Pará	Distrito Federal	Piauí
15	Mato Grosso do Sul	Ceará	Distrito Federal	Santa Catarina	Espírito Santo
16	Ceará	Piauí	Rio de Janeiro	Rondônia	Santa Catarina
17	Acre	Rondônia	Espírito Santo	Tocantins	Rio de Janeiro
18	Pará	Paraíba	Paraíba	Espírito Santo	Alagoas
19	Paraíba	Espírito Santo	Tocantins	Rio de Janeiro	Rondônia
20	Tocantins	Distrito Federal	Rondônia	Sergipe	Distrito Federal
21	Espírito Santo	Sergipe	Sergipe	Rio Grande so Sul	Rio Grande so Sul
22	Piauí	Alagoas	Alagoas	Alagoas	Rio Grande do Norte
23	Mato Grosso	Rio Grande do Norte	Rio Grande do Norte	Rio Grande do Norte	Tocantins
24	Rondônia	Amapá	Amapá	Amapá	Amapá
25	Amapá	Roraima	Roraima	Roraima	Roraima
26	Maranhão	Amazonas	Amazonas	Amazonas	Amazonas
27	Roraima	Acre	Acre	Acre	Acre

Table S3: List of the 27 Brazilian States (BS) sorted according to their associated metric under η_2. In the second column, the states are ordered according to the date of their first confirmed case.

	COVID-19	s	k	$\mathcal{V}$	b
1	São Paulo	São Paulo	Paraná	Goiás	Goiás
2	Rio de Janeiro	Paraná	São Paulo	Paraná	Mato Grosso
3	Bahia	Minas Gerais	Mato Grosso	Mato Grosso	Paraná
4	Distrito Federal	Santa Catarina	Minas Gerais	Tocantins	Tocantins
5	Minas Gerais	Mato Grosso	Goiás	São Paulo	São Paulo
6	Alagoas	Rio Grande so Sul	Mato Grosso do Sul	Bahia	Minas Gerais
7	Rio Grande so Sul	Bahia	Bahia	Minas Gerais	Bahia
8	Paraná	Mato Grosso do Sul	Tocantins	Mato Grosso do Sul	Mato Grosso do Sul
9	Santa Catarina	Goiás	Rio Grande so Sul	Rio de Janeiro	Distrito Federal
10	Goiás	Tocantins	Santa Catarina	Rio Grande so Sul	Rio Grande so Sul
11	Rio Grande do Norte	Pará	Rio de Janeiro	Santa Catarina	Santa Catarina
12	Pernambuco	Maranhão	Maranhão	Rondônia	Rio de Janeiro
13	Amazonas	Rio de Janeiro	Pará	Maranhão	Espírito Santo
14	Sergipe	Rondônia	Sergipe	Pará	Sergipe
15	Mato Grosso do Sul	Sergipe	Pernambuco	Sergipe	Alagoas
16	Ceará	Pernambuco	Paraíba	Pernambuco	Pernambuco
17	Acre	Paraíba	Rondônia	Paraíba	Paraíba
18	Pará	Distrito Federal	Distrito Federal	Distrito Federal	Rio Grande do Norte
19	Paraíba	Espírito Santo	Espírito Santo	Espírito Santo	Ceará
20	Tocantins	Alagoas	Alagoas	Alagoas	Piauí
21	Espírito Santo	Rio Grande do Norte	Rio Grande do Norte	Rio Grande do Norte	Maranhão
22	Piauí	Ceará	Ceará	Ceará	Amapá
23	Mato Grosso	Piauí	Piauí	Piauí	Pará
24	Rondônia	Amapá	Amapá	Amapá	Roraima
25	Amapá	Roraima	Roraima	Roraima	Amazonas
26	Maranhão	Amazonas	Amazonas	Amazonas	Acre
27	Roraima	Acre	Acre	Acre	Rondônia

Table S4: List of the first 30 cities from the Sao Paulo State (SP) sorted according to their associated metric under η_0. In the second column, the cities are ordered according to the date of their first confirmed case.

	COVID-19	s	k	$\mathcal{V}$	b
1	São Paulo	São Paulo	São Paulo	São Paulo	São Paulo
2	Santana de Parnaíba	Campinas	Campinas	São José do Rio Preto	Campinas
3	Ferraz de Vasconcelos	Ribeirão Preto	São José do Rio Preto	Ribeirão Preto	São José do Rio Preto
4	Mauá	São José do Rio Preto	Ribeirão Preto	Franca	Ribeirão Preto
5	Santo André	Peruíbe	Americana	Sorocaba	Presidente Prudente
6	São Bernardo do Campo	Sorocaba	Bauru	Taubaté	Bauru
7	São Caetano do Sul	Presidente Prudente	Piracicaba	Assis	Sorocaba
8	Guarulhos	Piracicaba	Marília	Penápolis	Piracicaba
9	Barueri	Jundiaí	Presidente Prudente	Piracicaba	Marília
10	Campinas	Americana	Santo André	Santa Fé do Sul	Araçatuba
11	Cotia	Bauru	Sorocaba	Apiaí	Americana
12	Jaguariúna	Marília	Araçatuba	Santa Isabel	Assis
13	Osasco	Araraquara	Araraquara	Pirapora do Bom Jesus	Catanduva
14	São José do Rio Preto	Mogi das Cruzes	São Bernardo do Campo	Presidente Prudente	Jales
15	São José dos Campos	Santa Isabel	São Carlos	Campinas	Taubaté
16	Suzano	Jaboticabal	Jaboticabal	Jales	São José dos Campos
17	Taubaté	São Carlos	Jundiaí	Rio Claro	Jundiaí
18	Vargem Grande Paulista	São José dos Campos	Rio Claro	Tupã	Franca
19	Hortolândia	Praia Grande	Osasco	Lins	Jaboticabal
20	Mogi das Cruzes	Rio Claro	Santos	Barretos	Santo André
21	Embu das Artes	Assis	Limeira	Araçatuba	Araraquara
22	Iracemápolis	Tupã	Assis	Osvaldo Cruz	Santos
23	Jundiaí	Taubaté	Tupã	Bauru	Itapetininga
24	Poá	Limeira	Catanduva	Catanduva	Ourinhos
25	Ribeirão Pires	São Vicente	Birigui	Monte Alto	Osasco
26	Rio Claro	Santos	Lins	Avaré	São Bernardo do Campo
27	São Sebastião	Araçatuba	Itapetininga	Capão Bonito	Barretos
28	Taboão da Serra	Itapetininga	São Vicente	Leme	Rio Claro
29	Valinhos	Registro	São José dos Campos	Bragança Paulista	Tupã
30	Arujá	Sertãozinho	Penápolis	Paraguaçu Paulista	Lins

Table S5: List of the first 30 cities from the Sao Paulo State (SP) sorted according to their associated metric under η_1. In the second column, the cities are ordered according to the date of their first confirmed case.

	COVID-19	s	k	$\mathcal{V}$	b
1	São Paulo	São Paulo	São Paulo	São Paulo	São Paulo
2	Santana de Parnaíba	Campinas	Campinas	Ribeirão Preto	São José do Rio Preto
3	Ferraz de Vasconcelos	Ribeirão Preto	São José do Rio Preto	São José do Rio Preto	Campinas
4	Mauá	São José do Rio Preto	Ribeirão Preto	Presidente Prudente	Ribeirão Preto
5	Santo André	Peruíbe	Presidente Prudente	Bauru	Presidente Prudente
6	São Bernardo do Campo	Presidente Prudente	Marília	Campinas	Marília
7	São Caetano do Sul	Mogi das Cruzes	Bauru	Franca	Bauru
8	Guarulhos	Sorocaba	Sorocaba	Avaré	Assis
9	Barueri	Santa Isabel	Araraquara	Marília	Lins
10	Campinas	Jundiaí	Assis	Registro	Registro
11	Cotia	Piracicaba	Jaboticabal	Itapetininga	Araraquara
12	Jaguariúna	Marília	Tatuí	Taubaté	Avaré
13	Osasco	Bauru	Piracicaba	Jaú	Sorocaba
14	São José do Rio Preto	São José dos Campos	Jundiaí	Araraquara	Catanduva
15	São José dos Campos	Araraquara	Itapetininga	Catanduva	Franca
16	Suzano	Americana	São José dos Campos	Assis	Araçatuba
17	Taubaté	São Carlos	Lins	Dracena	Ourinhos
18	Vargem Grande Paulista	Jaboticabal	Taubaté	Itapeva	Itapetininga
19	Hortolândia	Praia Grande	São Carlos	Lins	Jaboticabal
20	Mogi das Cruzes	Registro	Catanduva	Bragança Paulista	Jaú
21	Embu das Artes	Taubaté	Americana	Jundiaí	Jundiaí
22	Iracemápolis	Assis	Tupã	Bebedouro	Dracena
23	Jundiaí	Lorena	Registro	Barretos	Bebedouro
24	Poá	São Vicente	Porto Ferreira	São José dos Campos	São Roque
25	Ribeirão Pires	Itu	Parapuã	Jaboticabal	Taubaté
26	Rio Claro	Sertãozinho	Votuporanga	Atibaia	Itapeva
27	São Sebastião	Porto Ferreira	Limeira	Sorocaba	Piracicaba
28	Taboão da Serra	Limeira	São João da Boa Vista	Araçatuba	Atibaia
29	Valinhos	Rio Claro	Mogi das Cruzes	Santa Isabel	Tupã
30	Arujá	Araras	Lindóia	Pirapora do Bom Jesus	São José dos Campos

Table S6: List of the first 30 cities from the Sao Paulo State (SP) sorted according to their associated metric under η_2. In the second column, the cities are ordered according to the date of their first confirmed case.

	COVID-19	s	k	$\mathcal{V}$	b
1	São Paulo	São Paulo	São Paulo	São Paulo	São Paulo
2	Santana de Parnaíba	Campinas	Campinas	Bauru	Bauru
3	Ferraz de Vasconcelos	Ribeirão Preto	Ribeirão Preto	Marília	Marília
4	Mauá	Peruíbe	São José do Rio Preto	Ribeirão Preto	Ribeirão Preto
5	Santo André	Santa Isabel	Sorocaba	São José do Rio Preto	São José do Rio Preto
6	São Bernardo do Campo	São José do Rio Preto	Marília	Assis	Campinas
7	São Caetano do Sul	Mogi das Cruzes	Jundiaí	Miracatu	Assis
8	Guarulhos	Jundiaí	Taubaté	Campinas	Presidente Prudente
9	Barueri	Sorocaba	São José dos Campos	Sorocaba	Miracatu
10	Campinas	Piracicaba	Presidente Prudente	Jundiaí	Araraquara
11	Cotia	Praia Grande	Piracicaba	Santa Isabel	Sorocaba
12	Jaguariúna	São José dos Campos	Peruíbe	Atibaia	Santa Isabel
13	Osasco	Presidente Prudente	Lorena	Presidente Prudente	Registro
14	São José do Rio Preto	Lorena	Aparecida	Registro	Tupã
15	São José dos Campos	Taubaté	Tupã	Araraquara	Jacareí
16	Suzano	São Vicente	São Roque	Rio Claro	São Roque
17	Taubaté	Registro	Santa Isabel	Piracicaba	Jundiaí
18	Vargem Grande Paulista	São Roque	Rio Claro	Tupã	Rio Claro
19	Hortolândia	Itu	Registro	São Roque	Atibaia
20	Mogi das Cruzes	Rio Claro	Jaboticabal	Matão	Bragança Paulista
21	Embu das Artes	Araraquara	Guaratinguetá	Jacareí	Matão
22	Iracemápolis	Atibaia	Bauru	Bragança Paulista	Piracicaba
23	Jundiaí	Sertãozinho	Mogi das Cruzes	Mogi das Cruzes	Mogi das Cruzes
24	Poá	Aparecida	Jacareí	Araras	São Manuel
25	Ribeirão Pires	Guaratinguetá	Itu	Peruíbe	Parapuã
26	Rio Claro	Americana	Bragança Paulista	São Carlos	Araras
27	São Sebastião	São Carlos	Atibaia	Jaboticabal	Lençóis Paulista
28	Taboão da Serra	Pirapora do Bom Jesus	Araraquara	Franca	Aparecida
29	Valinhos	Jacareí	Sertãozinho	Jaú	Guaratinguetá
30	Arujá	Marília	São Vicente	São Manuel	Jaboticabal

Table S7: List of the first 30 Brazilian cities (BR) sorted according to their associated metric under η_0. In the second column, the cities are ordered according to the date of their first confirmed case.

	COVID-19	s	k	$\mathcal{V}$	b
1	São Paulo (SP)	São Paulo (SP)	São Paulo (SP)	São Paulo (SP)	São Paulo (SP)
2	Barra Mansa (RJ)	Belo Horizonte (MG)	Belo Horizonte (MG)	Belo Horizonte (MG)	Belo Horizonte (MG)
3	Feira de Santana (BA)	Salvador (BA)	Goiânia (GO)	Goiânia (GO)	Goiânia (GO)
4	Rio de Janeiro (RJ)	Aracaju (SE)	Campinas (SP)	Fortaleza (CE)	Brasília (DF)
5	Brasília (DF)	Campinas (SP)	Brasília (DF)	Porto Alegre (RS)	Rio de Janeiro (RJ)
6	Santana de Parnaíba (SP)	Goiânia (GO)	Rio de Janeiro (RJ)	Natal (RN)	Porto Alegre (RS)
7	Divinópolis (MG)	Fortaleza (CE)	Feira de Santana (BA)	Curitiba (PR)	Fortaleza (CE)
8	Maceió (AL)	Porto Alegre (RS)	Porto Alegre (RS)	Brasília (DF)	Campinas (SP)
9	Campo Bom (RS)	Campina Grande (PB)	Curitiba (PR)	São Luís (MA)	Curitiba (PR)
10	Porto Alegre (RS)	Feira de Santana (BA)	Salvador (BA)	Campina Grande (PB)	Natal (RN)
11	Caxias do Sul (RS)	Recife (PE)	Anápolis (GO)	Arapiraca (AL)	Feira de Santana (BA)
12	Cianorte (PR)	Caruaru (PE)	Cascavel (PR)	Patos (PB)	Salvador (BA)
13	Curitiba (PR)	Rio de Janeiro (RJ)	Ribeirão Preto (SP)	Rio de Janeiro (RJ)	Ribeirão Preto (SP)
14	Ferraz de Vasconcelos (SP)	Carpina (PE)	Fortaleza (CE)	Teresina (PI)	Teresina (PI)
15	Florianópolis (SC)	Teresina (PI)	Maringá (PR)	Aracaju (SE)	Recife (PE)
16	Goiânia (GO)	Ribeirão Preto (SP)	Carazinho (RS)	Palmas (TO)	Campina Grande (PB)
17	Ipatinga (MG)	Curitiba (PR)	São José do Rio Preto (SP)	Ribeirão Preto (SP)	São Luís (MA)
18	Natal (RN)	Brasília (DF)	Teresina (PI)	Ponte Nova (MG)	Anápolis (GO)
19	Niterói (RJ)	Limoeiro (PE)	Barreiras (BA)	São José do Rio Preto (SP)	João Pessoa (PB)
20	Recife (PE)	Paudalho (PE)	Campo Grande (MS)	Picos (PI)	Aracaju (SE)
21	Rio Verde (GO)	Belém (PA)	Vitória da Conquista (BA)	Manaus (AM)	São José do Rio Preto (SP)
22	Joinville (SC)	Patos (PB)	Uberlândia (MG)	Governador Valadares (MG)	Maceió (AL)
23	Manaus (AM)	São José do Rio Preto (SP)	Governador Valadares (MG)	Maceió (AL)	Maringá (PR)
24	Salvador (BA)	Castanhal (PA)	Recife (PE)	Rio Branco (AC)	Governador Valadares (MG)
25	Aracaju (SE)	Cascavel (PR)	Presidente Prudente (SP)	João Pessoa (PB)	Cascavel (PR)
26	Campo Grande (MS)	Maringá (PR)	São Leopoldo (RS)	Florianópolis (SC)	Florianópolis (SC)
27	Juiz de Fora (MG)	São Leopoldo (RS)	Cuiabá (MT)	Salinas (MG)	Carazinho (RS)
28	Patrocínio (MG)	Maceió (AL)	Florianópolis (SC)	Concórdia (SC)	Uberlândia (MG)
29	Rancho Queimado (SC)	Arapiraca (AL)	Londrina (PR)	Ijuí (RS)	Juiz de Fora (MG)
30	Braço do Norte (SC)	Maruim (SE)	Chapecó (SC)	Mossoró (RN)	Campo Grande (MS)

Table S8: List of the first 30 Brazilian cities (BR) sorted according to their associated metric under η_1. In the second column, the cities are ordered according to the date of their first confirmed case.

	COVID-19	s	k	$\mathcal{V}$	b
1	São Paulo (SP)	São Paulo (SP)	São Paulo (SP)	São Paulo (SP)	São Paulo (SP)
2	Barra Mansa (RJ)	Aracaju (SE)	Belo Horizonte (MG)	Belo Horizonte (MG)	Goiânia (GO)
3	Feira de Santana (BA)	Salvador (BA)	Goiânia (GO)	Goiânia (GO)	Recife (PE)
4	Rio de Janeiro (RJ)	Belo Horizonte (MG)	Porto Alegre (RS)	Teresina (PI)	Belo Horizonte (MG)
5	Brasília (DF)	Fortaleza (CE)	Salvador (BA)	Fortaleza (CE)	Feira de Santana (BA)
6	Santana de Parnaíba (SP)	Caruaru (PE)	Campinas (SP)	Recife (PE)	Teresina (PI)
7	Divinópolis (MG)	Campina Grande (PB)	Feira de Santana (BA)	Brasília (DF)	Brasília (DF)
8	Maceió (AL)	Recife (PE)	Curitiba (PR)	Natal (RN)	Petrolina (PE)
9	Campo Bom (RS)	Porto Alegre (RS)	Fortaleza (CE)	Campo Grande (MS)	Fortaleza (CE)
10	Porto Alegre (RS)	Feira de Santana (BA)	Teresina (PI)	São José do Rio Preto (SP)	Rio de Janeiro (RJ)
11	Caxias do Sul (RS)	Carpina (PE)	Rio de Janeiro (RJ)	Porto Alegre (RS)	Porto Alegre (RS)
12	Cianorte (PR)	Campinas (SP)	Cascavel (PR)	Araguaína (TO)	Curitiba (PR)
13	Curitiba (PR)	Goiânia (GO)	Ribeirão Preto (SP)	Feira de Santana (BA)	Cascavel (PR)
14	Ferraz de Vasconcelos (SP)	Rio de Janeiro (RJ)	Londrina (PR)	Campina Grande (PB)	Milagres (BA)
15	Florianópolis (SC)	Teresina (PI)	Recife (PE)	Vitória da Conquista (BA)	Carazinho (RS)
16	Goiânia (GO)	Limoeiro (PE)	São José do Rio Preto (SP)	Aracaju (SE)	Salvador (BA)
17	Ipatinga (MG)	Paudalho (PE)	Brasília (DF)	Rio de Janeiro (RJ)	Campinas (SP)
18	Natal (RN)	Ribeirão Preto (SP)	Cuiabá (MT)	Picos (PI)	Cuiabá (MT)
19	Niterói (RJ)	Patos (PB)	Aracaju (SE)	Arapiraca (AL)	Campo Grande (MS)
20	Recife (PE)	Castanhal (PA)	São Leopoldo (RS)	Curitiba (PR)	Aracaju (SE)
21	Rio Verde (GO)	Belém (PA)	Maringá (PR)	Juiz de Fora (MG)	Natal (RN)
22	Joinville (SC)	Maruim (SE)	Campina Grande (PB)	Maceió (AL)	São José do Rio Preto (SP)
23	Manaus (AM)	Rosário do Catete (SE)	Campo Grande (MS)	Rio Branco (AC)	Salgueiro (PE)
24	Salvador (BA)	Curitiba (PR)	Carazinho (RS)	Cascavel (PR)	Barreiras (BA)
25	Aracaju (SE)	Arapiraca (AL)	Belém (PA)	Mossoró (RN)	Maceió (AL)
26	Campo Grande (MS)	Laranjeiras (SE)	Caruaru (PE)	Manhuaçu (MG)	Governador Valadares (MG)
27	Juiz de Fora (MG)	Itabaiana (SE)	São Luís (MA)	São Luís (MA)	Ribeirão Preto (SP)
28	Patrocínio (MG)	Imperatriz (MA)	Presidente Prudente (SP)	Franca (SP)	Vitória (ES)
29	Rancho Queimado (SC)	Maceió (AL)	Juiz de Fora (MG)	Presidente Prudente (SP)	Campina Grande (PB)
30	Braço do Norte (SC)	São José do Rio Preto (SP)	Governador Valadares (MG)	Pau dos Ferros (RN)	Londrina (PR)

Table S9: List of the first 30 Brazilian cities (BR) sorted according to their associated metric under η_2. In the second column, the cities are ordered according to the date of their first confirmed case.

	COVID-19	s	k	$\mathcal{V}$	b
1	São Paulo (SP)	São Paulo (SP)	São Paulo (SP)	São Paulo (SP)	São Paulo (SP)
2	Barra Mansa (RJ)	Aracaju (SE)	Salvador (BA)	Salvador (BA)	Salvador (BA)
3	Feira de Santana (BA)	Salvador (BA)	Belo Horizonte (MG)	Goiânia (GO)	Goiânia (GO)
4	Rio de Janeiro (RJ)	Caruaru (PE)	Fortaleza (CE)	Belo Horizonte (MG)	Imperatriz (MA)
5	Brasília (DF)	Carpina (PE)	Porto Alegre (RS)	Aracaju (SE)	Cícero Dantas (BA)
6	Santana de Parnaíba (SP)	Campina Grande (PB)	Aracaju (SE)	Fortaleza (CE)	Paulo Afonso (BA)
7	Divinópolis (MG)	Recife (PE)	Campinas (SP)	Teresina (PI)	Jeremoabo (BA)
8	Maceió (AL)	Belo Horizonte (MG)	Feira de Santana (BA)	Porto Alegre (RS)	Serra Talhada (PE)
9	Campo Bom (RS)	Fortaleza (CE)	Campina Grande (PB)	Rio de Janeiro (RJ)	Recife (PE)
10	Porto Alegre (RS)	Limoeiro (PE)	Goiânia (GO)	Vitória (ES)	Teresina (PI)
11	Caxias do Sul (RS)	Paudalho (PE)	Caruaru (PE)	Cuiabá (MT)	Florianópolis (SC)
12	Cianorte (PR)	Feira de Santana (BA)	Recife (PE)	Salgueiro (PE)	Picos (PI)
13	Curitiba (PR)	Rio de Janeiro (RJ)	Rio de Janeiro (RJ)	Ribeirão Preto (SP)	Rio de Janeiro (RJ)
14	Ferraz de Vasconcelos (SP)	Campinas (SP)	Teresina (PI)	Picos (PI)	Feira de Santana (BA)
15	Florianópolis (SC)	Porto Alegre (RS)	São Leopoldo (RS)	Recife (PE)	Marcolândia (PI)
16	Goiânia (GO)	Maruim (SE)	Ribeirão Preto (SP)	Florianópolis (SC)	Araripina (PE)
17	Ipatinga (MG)	Rosário do Catete (SE)	Belém (PA)	Maceió (AL)	Belo Horizonte (MG)
18	Natal (RN)	Patos (PB)	São José do Rio Preto (SP)	Imperatriz (MA)	Aracaju (SE)
19	Niterói (RJ)	Teresina (PI)	Arapiraca (AL)	Campina Grande (PB)	Ouricuri (PE)
20	Recife (PE)	Castanhal (PA)	Marabá (PA)	Assis (SP)	Paraíso do Tocantins (TO)
21	Rio Verde (GO)	Laranjeiras (SE)	Rosário do Catete (SE)	São José do Rio Preto (SP)	Porangatu (GO)
22	Joinville (SC)	Arapiraca (AL)	Maruim (SE)	Propriá (SE)	Fortaleza (CE)
23	Manaus (AM)	Itabaiana (SE)	Maceió (AL)	Arapiraca (AL)	Salgueiro (PE)
24	Salvador (BA)	Goiânia (GO)	João Pessoa (PB)	Cascavel (PR)	Crato (CE)
25	Aracaju (SE)	Belém (PA)	Sobral (CE)	Marcolândia (PI)	Petrolândia (PE)
26	Campo Grande (MS)	Lagoa do Carro (PE)	Laranjeiras (SE)	Presidente Prudente (SP)	Floresta (PE)
27	Juiz de Fora (MG)	Ribeirão Preto (SP)	Patos (PB)	Araripina (PE)	Porto Alegre (RS)
28	Patrocínio (MG)	Vitória de Santo Antão (PE)	Castanhal (PA)	Curitiba (PR)	Milagres (BA)
29	Rancho Queimado (SC)	Lagoa Seca (PB)	Governador Valadares (MG)	Serra Talhada (PE)	Jequié (BA)
30	Braço do Norte (SC)	Escada (PE)	Imperatriz (MA)	Itabuna (BA)	Campina Grande (PB)
